# Oculocutaneous albinism and bleeding diathesis due to a novel deletion in the *HPS3* gene

**DOI:** 10.3389/fgene.2022.936064

**Published:** 2022-08-15

**Authors:** Dina Marek-Yagel, Shachar Abudi-Sinreich, Michal Macarov, Alvit Veber, Nechama Shalva, Amit Mary Philosoph, Ben Pode-Shakked, May Christine V. Malicdan, Yair Anikster

**Affiliations:** ^1^ Metabolic Disease Unit, Edmond and Lily Safra Children’s Hospital, Sheba Medical Center, Ramat Gan, Tel-Hahsomer, Israel; ^2^ Sackler Faculty of Medicine, Tel-Aviv University, Tel-Aviv, Israel; ^3^ Medical Genetics Branch, National Human Genome Research Institute, Bethesda, MD, United States; ^4^ Department of Ophthalmology, Hadassah-Hebrew University Medical Center, Jerusalem, Israel; ^5^ Talpiot Medical Leadership Program, Sheba Medical Center, Ramat Gan, Tel-Hahsomer, Israel; ^6^ NIH Undiagnosed Diseases Program, Common Fund, Office of the Director and National Human Genome Research Institute, Bethesda, MD, United States; ^7^ The Wohl Institute for Translational Medicine, Sheba Medical Center, Ramat Gan, Tel-Hahsomer, Israel

**Keywords:** Hermansky–Pudlak syndrome, HPS-3, *HPS3*, oculocutaneous albinism, Ashkenazi Jewish, deletion

## Abstract

Hermansky–Pudlak syndrome (HPS) is a group of rare autosomal recessive disorders characterized by oculocutaneous albinism (OCA) and bleeding diathesis. To date, 11 HPS types have been reported (HPS-1 to HPS-11), each defined by disease-causing variants in specific genes. Variants in the *HPS1* gene were found in approximately 15% of HPS patients, most of whom harbor the Puerto Rican founder mutation. In this study, we report six affected individuals from three nonconsanguineous families of Ashkenazi Jewish descent, who presented with OCA and multiple ecchymoses and had normal platelet number and size. Linkage analysis indicated complete segregation to *HPS3*. Sequencing of the whole coding region and the intron boundaries of *HPS3* revealed a heterozygous c.1163+1G>A variant in all six patients. Long-range PCR amplification revealed that all affected individuals also carry a 14,761bp deletion that includes the 5′UTR and exon 1 of *HPS3*, encompassing regions with long interspersed nuclear elements. The frequency of the c.1163+1G>A splice site variant was found to be 1:200 in the Ashkenazi Jewish population, whereas the large deletion was not detected in 300 Ashkenazi Jewish controls. These results present a novel *HPS3* deletion mutation and suggest that the prevalence of HPS-3 in Ashkenazi Jews is more common than previously thought.

## 1 Introduction

Oculocutaneous albinism (OCA) is a heterogeneous group of genetic disorders, caused by partial reduction or complete lack of melanin in melanocytes; it can be syndromic or nonsyndromic albinism. Nonsyndromic albinism is divided into X-linked ocular albinism (OA), which affects only the eyes, and OCA, which is variable hypopigmentation of the skin, hair, and eyes (Kamaraj and Purohit, 2014; Dolinska et al., 2017). Nonsyndromic OCA is a group of seven autosomal recessive disorders (OCA 1–7) caused by mutations in genes involved in melanin biosynthesis (Marcon and Maia, 2019). OCA1 (MIM 203100) is the most common subtype, caused by variants in the tyrosinase gene (*TYR*, MIM 606933), and is further divided into OCA1A, which occurs when tyrosinase activity is completely undetectable, and OCA1B, which has some residual enzyme activity (Gershoni-Baruch et al., 1994; Hutton and Spritz, 2008; Kamaraj and Purohit, 2014; Dolinska et al., 2017). OCA2 (MIM 203200) is caused by variants in *OCA2* (*P* gene); variants in *TYR* and *OCA2* genes account for most OCA cases (Yang et al., 2019).

Syndromic OCA includes genetic disorders that are characterized by hypopigmentation as a main clinical manifestation, accompanied by additional symptoms and often multisystemic involvement. These include autosomal recessive disorders such as Chediak–Higashi syndrome (MIM 214500), Griscelli syndrome (MIM 214450), and Hermansky–Pudlak syndrome (HPS, MIM 203300) (Kamaraj and Purohit, 2014; Marcon and Maia, 2019).

HPS is characterized by OCA and a bleeding diathesis. Hypopigmentation of the skin results in severe photosensitivity and predisposition to skin cancer. Ocular findings due to melanin pigment deficiency in the developing visual neural pathways result in the increased crossing of the optic nerve fibers, foveal hypoplasia, reduction in visual acuity, iris transillumination, nystagmus, and strabismus (Huizing et al., 1993; Marcon and Maia, 2019; Yang et al., 2019). The bleeding diathesis is caused by the absence of delta granules in platelets despite the normal numbers of platelets and can result in prolonged bleeding after minor procedures, variable bruising, epistaxis, gingival bleeding, and colonic bleeding. Other HPS manifestations include granulomatous colitis, neutropenia, and pulmonary fibrosis (Huizing et al., 1993; El-Chemaly and Young, 2016).

HPS is a rare disorder with a frequency estimated at 1–9 per 1,000,000. The disease is especially prevalent among the Puerto Rican population but is also found in Japan, India, Swiss, Ireland, South America, and Western Europe (Huizing et al., 1993; Huizing et al., 2020). To date, 11 types of HPS have been reported (HPS-1 to HPS-11), each defined by variants in a specific gene that encodes components of four protein complexes, namely, adaptor protein-3 and biogenesis of lysosome-related organelle complex-1–3 (BLOC-1–3), that impact membrane trafficking and protein sorting to facilitate lysosome-related organelle (LRO) maturation (Smith et al., 2005; Huizing et al., 2020; Pennamen et al., 2020). Most reported HPS patients are diagnosed with HPS-1 disease (MIM 604982), caused by pathogenic variants in the *HPS1* gene that encodes a component of the BLOC-3 complex (Gerondopoulos et al., 2012). Most HPS-1 patients are of Northwestern Puerto Rican ancestry because of a founder mutation of 16-bp duplication in *HPS1* (Santiago Borrero et al., 2006).

HPS-3 (MIM 614072) was first described in 2001 by Anikster *et al.* and is identified in approximately 15% of HPS patients (Anikster et al., 2001). There is a founder mutation in central Puerto Rico of a 3,904-bp deletion and three founder mutations in Ashkenazi Jews: c.1163+1G>A (also known as 1303+1G>A), 2621-2A>G and 1831+2T>G in *HPS3* (Anikster et al., 2001; Huizing et al., 2001; Torres-Serrant et al., 2010). HPS-3 protein is a component of BLOC-2, with HPS-5 and HPS-6 proteins; BLOC-2 might play a regulatory role in LRO biogenesis, as BLOC-2–deficient HPS patients lack the lung pathology observed in BLOC-3– and AP-3–deficient patients (Dennis et al., 2015). Moreover, HPS-3 is clinically mild compared with HPS-1, patients have milder OCA, and some HPS-3 patients were initially misdiagnosed with OA (Anikster et al., 2001; Torres-Serrant et al., 2010).

In this study, we report the clinical and mutational analyses of six HPS-3–affected individuals from three Israeli families of Ashkenazi Jewish descent.

## 2 Materials and methods

### 2.1 Patients

Written informed consent was obtained from the affected individuals or their legal guardians for genetic analysis and publication of patients’ photographs. Approval for human subject research was obtained from the Institutional Review Boards of the medical centers involved.

### 2.2 DNA and RNA purification from blood

Whole blood samples were taken from each participant into a 5-ml EDTA tube. DNA samples were extracted using the automatic device MagNa Pure LC system. RNA was extracted from blood samples using Trizol reagent according to the manufacturer’s instructions (Invitrogen, Carlsbad, CA). RNA was treated with DNA-free DNase to remove genomic DNA (Applied Biosystems, Austin, TX). Concentrations and purity were measured on the Nanodrop ND-1000 instrument (Nanodrop Technologies, Wilmington, DE). First-strand cDNA was synthesized using a high-capacity RNA-to-cDNA kit (Applied Biosystems, Austin, TX) according to the manufacturer’s guidelines.

### 2.3 Linkage analysis

Haplotypes were determined using five polymorphic markers from clones AC092979, AC021059059, AC131209, AC093001, and AC073522 located very close and on both sides of the gene. The markers were amplified with fluorescently labeled primers (Supplementary Table S1) in the condition described below. The PCR fragments were separated using the automated ABI Prism 3100 Genetic Analyzer (Perkin Elmer) and analyzed using Peak Scanner Software (Thermo Fisher Scientific).

### 2.4 Sequencing and mutation analysis

DNA and cDNA amplification was conducted in a 25-µl reaction containing 50 ng of DNA, 10 pmol of each primer, ddH2O, and Red Load Taq Master*5 (LAEOVA). After an initial denaturation of 5 min at 95°C, 35 cycles were performed (94°C for 30 s, 56–60°C for 30 s, and 72°C for 30 s), followed by a final extension of 10 min at 72°C. Primer pairs for amplifying each *HPS3* exon on genomic DNA have been described in previous work (Shotelersuk et al., 1998). cDNA amplification for the c.1163+1G>A mutation was conducted using primers flunking 600 bp from the cDNA sequence (including exon 5) of the *HPS3* gene, to obtain 400 bp for the mutant allele and 600 bp for the normal allele (Supplementary Table S2). Sequencing was performed at the molecular biology service lab, Hy-labs (Rehovot, Israel).

Long-range PCR was utilized to map the putative deletion boundaries. PCR primers were designed by a walking strategy, and the resulting amplification products were sequenced. The deletion was first detected with primers conducting 3000-bp product only with the mutant allele (Supplementary Table S2). Long-range PCR was conducted in a 50-µl reaction by using Advantage®-GC 2 PCR kit (Clontech), according to the manufacturer’s instructions. Sequencing was performed using an automated ABI Prism 3100 Genetic Analyzer (Perkin Elmer). The deletion in the families was demonstrated by primer sets located across the deletion and inner reverse primer within the deletion (Supplementary Table S2). Carrier rates were established using restriction enzyme assay with *RsaI* (Oh et al., 1996) for the splice mutation in 1000 unaffected Ashkenazi Jewish population. Multiplex reverse-transcription PCR (RT-PCR) of 300 cDNA samples from unaffected Ashkenazi Jewish individuals was used to determine the frequency of the *HPS3* deletion (Supplementary Table S2).

### 2.5 Cell culture

Patient primary fibroblasts were cultured from a forearm skin biopsy. Control fibroblasts were purchased from ATCC (Manassas, VA). Fibroblasts were grown in a high-glucose (4.5 g/L) DMEM medium supplemented with 10% fetal bovine serum (Gemini Bio-Products, West Sacramento, CA) and penicillin-streptomycin (Thermo Fisher Scientific Cat 15070063). Cells were grown in 75-cm^2^ flasks. When fully confluent, cells were washed twice with PBS and trypsinized with TrypLE (Thermo Fisher Scientific, Cat 12563011). Cell lysates were collected, washed twice with PBS, and centrifuged to obtain cell pellets (20,000 × g, for 15 min at 4°C) and then frozen at −80°C.

### 2.6 Real-time quantitative PCR

RNA was extracted from the frozen cell pellet using the RNeasy maxi kit (QIAGEN, Cat 75,162) according to the manufacturer’s protocol. RNA was treated using a DNA-free DNA removal kit (Invitrogen, Cat AM1906) and transcribed into cDNA using an high-capacity RNA-to-cDNA kit (Applied Biosystems, Cat 4387406). RNA concentration was measured using a Nanodrop ND-1000 spectrophotometer (Thermo Fisher Scientific). Relative mRNA expression of *HPS3* was measured using TaqMan Universal PCR Master Mix (Applied Biosystems, Cat 4305719) and the following TaqMan probes, namely, *HPS3*, *HPRT1* (Thermo Fisher Scientific, Hs00289968_m1, 4333768T), using the QuantStudio 6 Pro Real-Time PCR System (Thermo Fisher Scientific). Results were normalized with the expression of the housekeeping gene *HPRT1*.

### 2.7 Protein quantification

Proteins were isolated using RIPA buffer (Sigma-Aldrich, Cat R0278-50 ML) containing a protease inhibitor cocktail (Thermo Fisher Scientific, Cat 78,430). The total amount of protein in each sample was determined using the BCA assay kit (Thermo Fisher Scientific, Cat 23,235). Protein lysates were reduced with LDS sample buffer (Invitrogen, Cat B0007) supplemented with a reducing agent (Invitrogen, Cat B0009) and electrophoresed on 8% Bis-Tris Bolt gel (Invitrogen, Cat NW00080BOX) with MOPS buffer (Invitrogen, Cat B0001) and antioxidant (Invitrogen, BT0005). The molecular weight ladder Prestained Protein Standards Kaleidoscope (Bio Rad, Cat 1610375) was used to ascertain protein molecular weight. Proteins separated on the gel were transferred to a PVDF membrane (Invitrogen, Cat IB24002), blocked with 5% nonfat milk for 1 h, and incubated overnight at 4°C with primary anti-HPS-5 rabbit polyclonal antibody (Proteintech, Cat 13901-1-AP) and anti-beta Actin mouse monoclonal antibody (Abcam, Cat mAbcam 8226). After three washes (5 min) in TBS with 0.1% Tween-20 (TBST), the membrane was incubated with the appropriate secondary antibodies (IRDye 800CW Cat. 926-32213, IRDye680RD Cat. 926-68072, Li-Cor Biosciences). The membrane was then washed (5 min, three times), and the target bands were imaged under IR Odyssey Imaging System (LiCOR, Lincoln, NE).

## 3 Results

### 3.1 Clinical presentations

Six affected individuals from three families of Ashkenazi Jewish descent were evaluated at the Metabolic Disease Unit at the Edmond and Lily Safra Children’s Hospital, Sheba Medical Center, Israel.

Family A includes two male siblings (probands 1 and 2) who were referred to Sheba Medical Center at the ages of 4 and 3 years. They were born to nonconsanguineous parents of Ashkenazi Jewish descent. Two additional siblings, an older brother (5 years old) and a younger sister (1 year old), were reportedly healthy. The patients presented with albinism, nystagmus, and a tendency toward hematomas following physical activity. Cognitive development seems to be normal. They were not available for further phenotyping as they are currently followed up elsewhere.

Family B includes proband 3 who was born to nonconsanguineous parents of Ashkenazi Jewish descent, with two healthy older siblings. He was born by vaginal delivery following an uneventful pregnancy, at a birth weight of 3600 g. The proband was circumcised on the eighth day of life, without significant bleeding. At the age of 6 weeks, the mother had noticed nystagmus, prompting consult, with a recommendation to continue follow-up at the age of 6 months. At the age of 5 months, he was diagnosed with albinism and first seen by a clinical geneticist for evaluation of suspected OCA. The common mutations for OCA1 and OCA2 were evaluated and ruled out. At 1.5 years of age, proband 3 required ophthalmologic surgery to correct strabismus. Proband 3 was referred to Sheba Medical Center at the age of 2.5 years for further evaluation. Upon examination, he showed a cognitive developmental status within normal limits, albinism, and several ecchymoses on his forehead, back, and upper and lower limbs. His height and weight percentiles were approximately 25% and 55%, respectively. His initial laboratory workup had shown a complete blood count within normal limits (WBC 10.5 k/µl, hemoglobin 12.8 g/dl, platelets 276 K/µl, MPV 9.6 fL) and an INR of 1.01 (PT 11 s, aPTT 33.6 s). His family history revealed that the mother had two cousins (male and female) who were also suspected of OCA. His mother and father, however, had brown and blonde hair, respectively, and had shown no additional signs of OCA or HPS.

**FIGURE 1:**
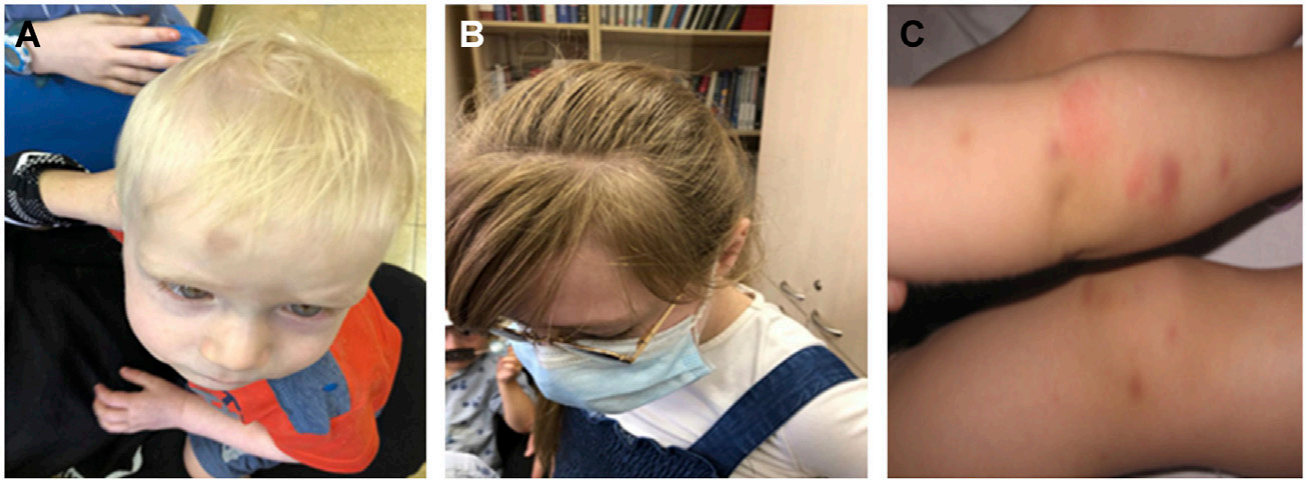
Clinical manifestations of Hermansky–Pudlak syndrome (HPS)-3 patients. HPS-3 patients have variable hypopigmentation of skin and hair, as shown in probands 4 and 5 (B-II:8 and B-II:9, respectively) **(A–B)**, and some show multiple ecchymoses due to a bleeding diathesis in HPS patients (proband 5) **(C)**.

Affected individuals in family C were first clinically diagnosed at the Michelson Institute (Hadassah Medical Center, Jerusalem, Israel), and then referred to our clinic for molecular analysis. Of the five siblings, three are affected (probands 4, 5, and 6), including two brothers at the ages of 1.5 and 9 years and one sister at the age of 11. Two additional male siblings (7 and 4 years old) were reportedly healthy. The three affected siblings exhibit hypopigmentation of the skin and the hair, nystagmus, and a tendency to bleed. Of note, with time, an increase in pigmentation was observed in the older sister (clinical evaluation shown in Figure 1).

### 3.2 Molecular diagnosis

**FIGURE 2:**
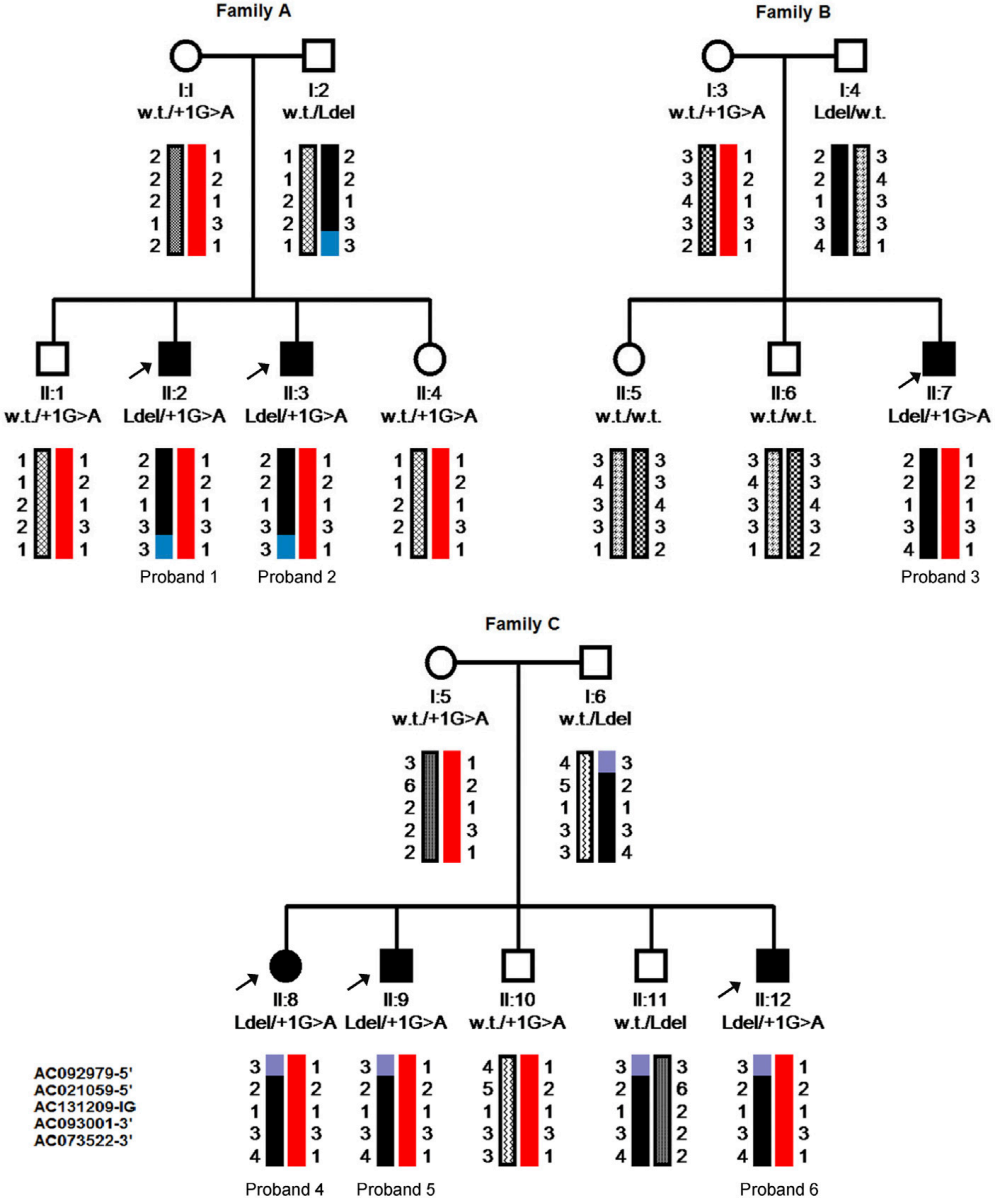
Pedigrees and Haplotype analysis of the six probands’ families. Pedigrees include genotyping results shown as wild type (w.t.), large deletion (Ldel) or splice site mutation (+1G>A), and the inherited chromosomes; each allele illustrated with different patterns. Five polymorphic markers from clones located very close and on both sides of the *HPS3* gene were used. From top to bottom: AC092979, AC021059059 (5′-upstream of the *HPS3* gene), AC131209 (Intra genic-IG), AC093001, AC073522 (3′-downstream of the gene). Arrows pint to the affected member in each family.

Patients from family B were first tested for common mutations in genes associated with OCA (c.IVS2-7T>A variant in the *TYR* gene known to cause OCA1; p.G27R, p.A481T, and p.V443I mutations in the *P* gene associated with OCA2) but were normal. Sequencing of the whole coding region and the intron boundaries of *HPS3* revealed the known c.1163+1G>A mutation, which is predicted to result in the skipping of exon 5, in a heterozygous state in all six affected individuals. Complete sequencing of the entire gene, including all intronic regions, did not reveal additional pathogenic variants in the gene. Thus, the corresponding genomic regions were screened for the presence of microsatellite repeats for haplotype analysis. The patients and their families were screened using five polymorphic markers from five different clones: AC092979 and AC021059059 located upstream to the gene, AC131209 located at the gene clone, and AC093001 and AC073522 located downstream to the gene. Haplotype analysis revealed full segregation in the families, confirming that all probands share the same heterozygous haplotype (Figure 2).

**FIGURE 3:**
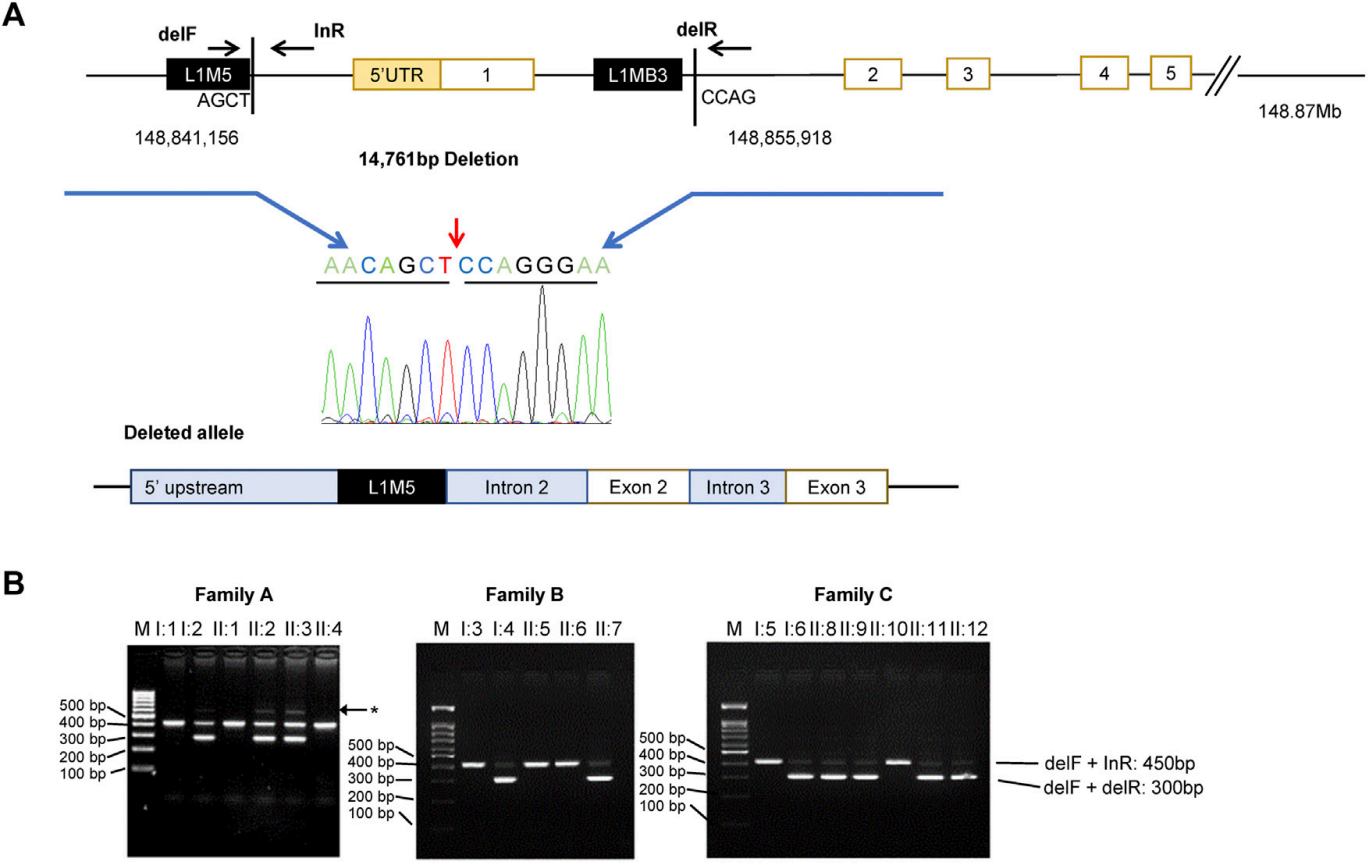
Identification of a large deletion in *HPS3*. **(A)** Top: Scheme of the *HPS3* deletion and multiplex PCR amplification assay with three primers: delF and delR across the deletion and inR within the deletion. Chromatogram of the deletion breakpoint sequence. Bottom: Schematic basis of the deleted allele, which shows deletion of the 5′ UTR and exon 1. The deletion occurs between two LINE-1 repeats (black box). **(B)** Multiplex PCR amplification yield a lower 300 bp band (del1F + delR) and upper 450 bp band (delF + inR) for the normal and mutant allele, respectively. Asterisk denotes nonspecific bands (faint band ∼600 bp). M, marker (100 kb ladder); bp, base pairs.

Since previous reports have shown *HPS3* deletions in exon 1, long-range PCR amplification of an area encompassing the 5′UTR and exon 1 (ch3:148841157-148855918; GRCh37) was performed and followed using Sanger sequencing. A deletion of 14,761 bp was detected, encompassing the entire 5′UTR (86 bp), a region upstream exon 1 (6268 bp), all of exon 1 (300 bp), and part of intron 1 (8193 bp) (Figure 3A). Multiplex PCR amplification assay was also designed to detect the deletion in the families. The PCR was conducted by primers flanking the deletion, resulting in a ∼450-bp PCR product representing the normal allele and a ∼300-bp amplicon representing the deleted allele (Figure 3B). All probands were found to be heterozygous for the deletion that was fully segregated with the disease in the families. Bioinformatic analysis and inspection of the deletion region in UCSC Browser (https://genome.ucsc.edu/) and Repeat masker (https://www.repeatmasker.org/) showed a number of long interspersed nuclear elements (LINEs) and Alu repeats surrounding the deletion. The 5′ of the deletion starts at the last nucleotide of LIM5, which is located 200 bp from the L1MB3 in the 3′ end (Figure 3A).

Next, cDNA from blood-derived RNA from affected individual II:7, his mother (I:3), and healthy controls were synthesized. cDNA amplification was conducted with primers flanking exon 5 and resulted in two fragments, namely, 600 bp in the normal allele and 400 bp in the mutant allele (without exon 5). The patient exhibited a mutant fragment and a very faint wild-type band. An allele-specific amplification for the splice mutated allele was conducted with a forward primer located inside exon 1 (Supplementary Table S2), which allowed amplification of the two fragments.

### 3.3 *HPS3* expression and BLOC-2 stability in fibroblasts

**FIGURE 4:**
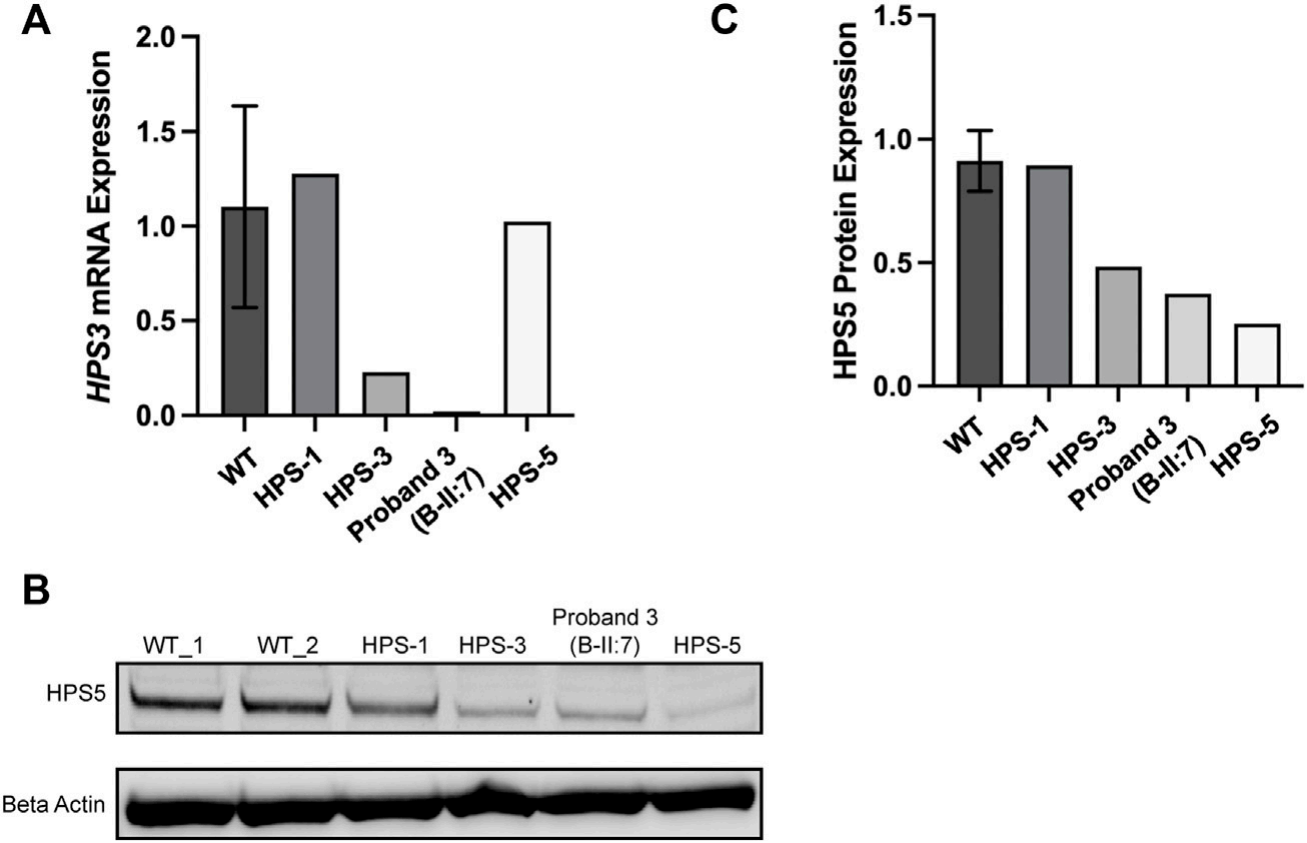
Molecular analysis of the compound heterozygous probands. **(A)** *HPS3* mRNA expression in fibroblasts from four different WT controls, Hermansky–Pudlak syndrome (HPS)-1 (patient with biallelic mutations in *HPS1*), HPS-3 (patient with biallelic mutations in *HPS3*), and HPS-5 (patient with biallelic mutations in *HPS5*). Proband 3 is B-II:5 and with HPS-3 disorder. *HPS3* mRNA expression is reduced in patients with biallelic mutations in *HPS3* as compared with control. **(B)** Western blotting from the mentioned above samples probing for HPS-5 aiming to evaluate BLOC-2 stability; β (beta) Actin was used as a control to normalize results. **(C)** Western blotting quantification of B, showing that HPS-5 protein is decreased in HPS-3 patient, proband II:5) to approximately 40% compared with WT.

RNA and proteins were extracted from fibroblasts of proband II:5 and controls including normal control, other BLOC-2 partners (known HPS-3 and HPS-5 patients), and unaffected BLOC-3 mutant (HPS-1 patient). RT-PCR shows a significant decrease in *HPS3* mRNA expression in patient II:5. No significant change was observed in neither HPS-1 nor HPS-5 patients when comparing expression to four different wild-type controls (Figure 4A). To evaluate BLOC-2 stability when the *HPS3* level is reduced, a western blotting with an anti-HPS-5 antibody was conducted with proteins extracted from samples of patient II:5 and the same controls. The results show a faint band in HPS-5 patients and a decrease to approximately 25% expression compared with unaffected control (WT) and approximately 40% expression of HPS-5 protein in HPS-3 patients, proband II:5. However, BLOC-3 stability was not affected (Figures 4B,C).

### 3.4 *HPS3* mutations in the Ashkenazi Jewish population

To assess the frequencies of these disease-causing variants in the Ashkenazi Jewish population, a restriction enzyme assay was utilized for the c.1163+1G>A splice site variant, which creates an *RsaI* restriction site in the carrier chromosomes. A carrier rate of 1:200 was found in Ashkenazi Jewish controls (data are not shown). The deletion frequency was tested by the multiplex primers in over 300 Ashkenazi Jewish controls; however, no carriers were found. Moreover, the deletion was not found in over 3000 Chromosomal Microarray Analysis tests of the Israeli population, and it is not described in the CNV databases.

## 4 Discussion

Here, we report six affected individuals from three Ashkenazi Jewish families with OCA and bleeding tendencies. All probands had no additional symptoms that could raise the suspicion of HPS, such as immunodeficiency, granulomatous colitis, or pulmonary fibrosis. Extensive genetic analysis revealed compound heterozygous variants in *HPS3*, the previously described c.1163+1G>A variant, and a novel large deletion.

The deletion encompassed all of exon 1, 6268 bp upstream to exon 1, and 8193 bp from intron 1. It is possible that the deletion arose from the two mobile elements, namely, LIM5 and LIMB3, which could have engulfed the deleted area. These elements belong to Long Interspersed Nuclear Element-1s (LINE-1s), which are the only active endogenous transposons in the human genome. These elements comprise approximately 15% of the genome, with more than 100,000 copies that have been amplified during the last 1000 million years of mammalian evolution (Goodier, 2016). Most of the LINE-1s are capable of retro-transposition, and some of the young elements inserted into the human genome recently, resulting in variability in the LINE chromosomal location. The consensus human LINE contains two active open reading frames (ORF1 and ORF2) separated by a short sequence (Feng et al., 1996). The deletion between the LINEs may occur by homologous recombination between the two elements, or retro-transposition of one or two of them. *HPS3* 5′ UTR and exon 1 domain harbor multiple repetitive elements from SINE and LINE families. We have previously reported a ∼4000-bp founder deletion in Puerto Rico (Anikster et al., 2001) that overlaps with the novel deletion and occurs between two Alu repeats (AluYa5 and AluSg) from the SINE family, including all of exon 1 and ∼3000 bp and ∼700 bp from the upstream exon 1 and intron 1, respectively.

Splicing-related mutations may have various effects on RNA processing, such as exon skipping, intron inclusion, cryptic splicing, and leaky splicing. The c.1163+1G>A splice site variant was first described in 2001 (Huizing et al., 2001) as a common mutation in the Ashkenazi Jewish population that leads to the skipping of exon 5. Expression analysis of *HPS3* in patient II:7 showed that *HPS3* mRNA levels decreased, but it was not completely absent. Moreover, the mRNA product from the splice mutated allele was amplified, where a faint PCR product was observed. These indicated a leaky splice site, which causes incomplete inhibition of the normal splice site resulting in a small production of the normal allele, which might explain the mild phenotype of the patients. The “leakiness” of the mutation may support therapeutic intervention with splice switching oligonucleotides, nucleic acids that are designed to create a steric block to the binding of splicing factors to the pre-mRNA, which leads to an alteration of normal splicing of the targeted transcript (Havens and Hastings, 2016).

The frequency of the splice mutation was previously estimated to be 1 in 235 healthy Ashkenazi Jewish individuals (Huizing et al., 2001). However, in this study, screening of 1000 controls revealed a higher carrier rate of 1 in 200 in this subpopulation. Haplotype analysis of the three families revealed different founder alleles for each mutation, with ∼400 Mbp shared haplotype (from AC021059 to AC093001), suggesting a common founder allele. According to the frequencies of the two mutations, the c.1163+1G>A mutation is more ancient.

Overall, the estimated prevalence of OCA in the Israeli population is approximately 1 in 10,000 and is caused by several genes including mainly *TYR*, *P*, *HPS1*, and *HPS3*, and even some mutations in *HPS4* were described in a patient of Ashkenazi Jewish origin (Rosenmann et al., 2009; Lozynska et al., 2018). Founder mutations and additional variants in HPS genes including the newly identified large deletion reported herein in the Ashkenazi Jewish descent underscore the diagnostic possibility of HPS among individuals affected with albinism in Israel. Screening for founder mutations and the *HPS3* large deletion mutation should be considered in relevant clinical circumstances.

This work emphasizes the importance of screening for HPS mutations in populations prone to have founder mutations to avoid misdiagnosis of HPS. HPS patients have a higher risk of developing bleeding disorders, granulomatous colitis, neutropenia, and, most importantly, HPS-associated pulmonary fibrosis, and because of early intervention programs, a correct and timely diagnosis is crucial.

## Data Availability

The data sets for this study are available upon reasonable request from the corresponding author. The data are not publicly available due to privacy or ethical restrictions.
