# Supplementary material for: Oculocutaneous albinism and bleeding diathesis due to a novel deletion in the HPS3 gene
Source: Front Genet. 2022 Aug 15;13:936064. doi: 10.3389/fgene.2022.936064 (PMC9420964; doi:10.3389/fgene.2022.936064)
Supplement: Supplementary file 1 [file Table1.DOCX]

Supplementary Material

## Supplementary Tables

| **Reverse 5'-3'** | **Forward 5'-3'** | **Primer Name** |
| --- | --- | --- |
| CCTGCTGACATTTTGATTTGG | TTAATCCCCCATTGCTCAAC | AC092979 |
| AACATGGCAAAACCGTGTCT | GCCTCAGCCTCCTGATGAGT | AC021059 |
| ATTTCCAGTTCCTGGGTCCT | AATCCTCTGTTCCAGCTCCA | AC131209 |
| GGCCCCACAAATGTTGAGTA | CACAACCAAGGCTCTTCCAT | AC093001 |
| GGTTTTTCTGGAGCTAGATTTCA | ACGTTGTCTCATGCAGCAGT | AC073522 |

**Supplementary Figure 1. Polymorphic markers primers list.**

| **Reverse 5'-3'** | **Forward 5'-3'** | **Primer Name** |
| --- | --- | --- |
| TGTATTCTTAAAGCACAGACATCCA | TCGAATAAGACGGACAGAAGAA | HPS3_cDNA |
| CTGGCCATCTTTTCCTACGTTT | TCTTCTCTGCATTGTATTTTTGGTGT | Deletion boundaries |
| GGTTCAGGCTCCATCTCT | CTTTTTGAGTGAGCTTTGGTAGC | Across the deletion (delF+delR)-mutated allele) |
| CATCTTCCCCCATTCTGAGA | CTTTTTGAGTGAGCTTTGGTAGC | Within the deletion (delF+inR)-wide type allele |

**Supplementary Figure 2. cDNA amplification primers list.**
